# Pheochromocytoma Masquerading as “Diabetic Ketoacidosis”

**DOI:** 10.1177/2324709616646128

**Published:** 2016-04-21

**Authors:** Jeffrey Stephen Hedley, Sidney Law, Sujoy Phookan, Maria Nien-Feng Lee, Adriana Ioachimescu, Rebecca D. Levit

**Affiliations:** 1 Emory University, Atlanta, GA, USA

**Keywords:** pheochromocytoma, diabetic ketoacidosis, DKA, stress hyperglycemia, stress response

## Abstract

Diabetic ketoacidosis is a routinely encountered diagnosis in medicine. Physicians are trained early on to look for precipitants. Most clinicians assess for medication compliance, infection, ischemia, and the like. We present a case of pheochromocytoma presenting as “diabetic ketoacidosis.” The case serves as an example for broadening the differential diagnosis for patients with similar presentations. Additionally, the case helps inform our understanding of the so-called “stress reactions” that are commonly invoked in clinical rationale.

## Presentation

A 62-year-old Caucasian gentleman presented to the emergency department complaining of palpitations. The patient, an avid ice hockey player, was participating in a game the evening prior to admission. After a long stretch of play, he began to experience headache, lightheadedness, and palpitations. The patient reported feeling shaky and weak, and although he is was not a known diabetic, he postulated that his symptoms might be consistent with those of low blood glucose. He drank several cans of soda with some alleviation of his complaints. His symptoms persisted throughout the night, and by early next morning, he developed dry heaves and nausea, which progressed to diaphoresis and finally chest pain. At that point the patient drove himself to the emergency department for evaluation.

The patient’s medical history is significant only for well-controlled hypertension on amlodipine 10 mg and metoprolol succinate 25 mg daily, and a left ureteral stent and lithotripsy 10 years ago for a ureteral stone. He took a daily statin and aspirin but no other medications and denied allergies. He did not smoke, drink alcohol in excess, or use any illicit drugs. Family history was remarkable for a brother with coronary artery disease and myocardial infarction. He worked locally as a database administrator.

He experienced similar episodes of palpitations 1 year ago and saw a physician for evaluation. A nuclear stress test was performed at another facility and was reportedly negative. He denied prior use of a monitoring device to identify occult arrhythmias.

On examination, the temperature was 36.6°C, pulse was 111 beats per minute and regular, blood pressure was 194/110 mm Hg, respiratory rate was 19, and oxygen saturation was 95% on room air. No enlargement, nodules, or tenderness of the thyroid were appreciated. On examination of the heart, point of maximal impulse was neither enlarged nor displaced. S1 and S2 were heard without a gallop, rub, or murmurs. Lungs were clear to auscultation bilaterally and abdominal exam unremarkable. The rest of the physical examination was normal.

Basic chemistries performed on admission were remarkable for the following: potassium 3.0 mEq/L (reference: 3.5-5.0), bicarbonate 17 mmol/L (18-23) with anion gap of 21 mmol/L (2-11), elevated creatinine of 2.06 mg/dL (0.8-1.3) (unknown baseline) with normal blood urea nitrogen, low inorganic phosphorous of 1.3 mg/dL (3.0-4.5), and elevated serum glucose 378 mg/dL (65-110). Transaminases, alkaline phosphatase, bilirubin, total protein, and albumin were all normal. Serum β-hydroxybutyrate was normal though urinalysis had elevated ketones at 10 mg/dL (normal is negative) and greater than 1000 mg/dL of glucose (normal is negative). Hemoglobin A1c was 5.7% (4.3% to 6.1%). Thyroid studies were obtained and within normal limits. Parathyroid hormone was mildly elevated at 83 pg/mL (14-72), and calcium was normal at 9.8 mg/dL (8.9-10.3). An ABG performed on non-rebreather (several hours after admission) with 100% FiO_2_ showed pH of 7.44, CO_2_ 30 mm Hg, and PaO_2_ 51 mm Hg, and a lactic acid level of 2.76 mmol/L (0.51-2.2). Complete blood count was remarkable for white blood cell count of 21 000/µL (4200-9100), hemoglobin of 17.8 g/dL (12.9-16.1), and mildly elevated mean corpuscular volume of 94 fL (79-92.2). Platelets were within normal limits. Troponin was elevated initially to 0.83 ng/mL, subsequently rising to 2.69 ng/mL in 6 hours (<0.04). Brain natriuretic peptide was elevated at 421 pg/mL (<99).

Initial chest X-ray was normal. Electrocardiogram showed mild ST elevation in leads aVR as well as diffuse ST depressions in leads V2-6 and I, II, and aVF. A transthoracic echocardiogram performed on admission demonstrated the following: left ventricular ejection fraction 55% to 60%; no regional wall motion abnormalities, restrictive filling, mild mitral, and tricuspid valve regurgitation; and normal right ventricular size, function, and wall thickness.

At this point the patient was treated as hypertensive emergency in light of elevated blood pressure and non-ST segment elevation myocardial infarction (NSTEMI). A nitroglycerin drip was initiated with adequate and safe control of blood pressure. The patient was given aspirin, a clopidogrel loading dose, heparin infusion, and β-blocker. During this time the patient remained free of chest pain.

Given the hyperglycemia and elevated anion gap, there was concern for diabetic ketoacidosis (DKA) in the emergency department. The patient was treated in the usual fashion with an insulin drip and intravenous fluids (IVF). However, the patient was soon noted to have increasing oxygen requirements, which were secondary to pulmonary edema from IVF. Bilevel positive airway pressure was required and the patient was admitted to the coronary care unit.

In the coronary care unit, the patient was diuresed with resolution of the pulmonary edema and increased oxygen requirements. Nitroglycerin drip was weaned off as adequate blood pressure control was achieved with oral medications. A left heart catheterization on hospital day 2 revealed only nonobstructive coronary artery disease with a 40% to 50% lesion of the mid-LAD (left artery descending) with an fractional flow reserve of 0.89 (<0.75 or 0.8 is significant).

As expected, telemetry soon revealed paroxysmal atrial fibrillation with rapid ventricular rate of roughly 140 beats per minute. A bolus, followed by continuous infusion of amiodarone, was initiated with excellent rate control and, briefly, chemical cardioversion to sinus rhythm.

Given the stability of the patient, he was transferred to the general academic cardiology service. Soon after arrival on the floor, the patient experienced dramatic increase in blood pressure (240/130 mm Hg). At this point, his chest pain returned but was now rated at a 10 on a scale of 1 to 10. He was again transferred to an intensive care unit where he was initiated on a nicardipine drip. Diagnostic testing was performed at this time.

## Diagnostic Discussion

From the beginning, an occult arrhythmia was naturally expected given the history of paroxysmal palpitations. The initial impression was that this occult tachyarrhythmia had precipitated an NSTEMI in a type II fashion. This, in turn, prompted a stress response that manifested itself in the form of leukocytosis and hyperglycemia. While arrhythmias with rapid heart rate certainly increase cardiac oxygen demand, the degree of blood pressure elevation is unexpected and implies another stimulus not yet realized.

The original diagnosis of DKA was quickly abandoned due to lack of elevated β-hydroxybutyrate, which prompted the team of providers to look for another etiology of rapid fluctuation from hypoglycemia to hyperglycemia in a patient with no history of diabetes. A strong hyperglycemic response to stress (infection, ischemia) is known to occur with some patients and confers increased risk for future development of diabetes. But that is not enough to explain what was perceived to be an episode of hypoglycemia on the night prior to admission.

Perhaps most telling was the patient’s dramatic rise in blood pressure once having been transferred to the floor in what was previously a completely stable clinical condition.

## Hospital Course

A computed tomography of the chest and abdomen with IV contrast was ordered to rule out aortic dissection. No dissection was seen but a right adrenal mass measuring 4.6 cm in diameter was incidentally visualized. This same mass was noted to be 2 × 2.3 cm during evaluation of his previous ureteral stone 10 years ago. Follow-up abdominal magnetic resonance imaging demonstrated a 4.7 × 4.7 cm mass of the right adrenal gland. Given the rate of growth and the clinical scenario, the adrenal mass was suspected to be a pheochromocytoma. Plasma and urine metanephrines were ordered and noted to be elevated to 4.65 nmol/L (<0.50) and 2498 µg/24 h (<400), respectively. Additionally, plasma and urine normetanephrines were 15 061 pg/mL (80-520) and 5117 µg/24 h (<900), respectively. Urine dopamine was 673 µg/24 h (65-100). The endocrinology service and general surgery services were consulted. The patient was started on phenoxybenzamine, as well as carvedilol and amlodipine, with successful control of blood pressure. For the atrial fibrillation, he was continued on amiodarone and anticoagulated with apixaban with anticipation that after successful removal of the tumor his arrhythmia would terminate.

## Follow-up Course

One month after his hospital course, the patient underwent a successful laparoscopic right adrenalectomy without complications and was discharged the following day. Amiodarone, carvedilol, and apixiban were discontinued. On postoperative day 12 he was seen in cardiology clinic where the remainder of his antihypertensives were discontinued and started on atenolol 50 mg daily. Recheck of plasma metanephrines on postoperative day 15 showed that they had returned to normal levels. Aside from one episode of palpitations on postoperative day 1, the patient remained symptom free at 4-month follow-up. To date, no genetic testing such as investigation into multiple endocrine neoplasia syndromes has been performed.

## Review

Pheochromocytomas are catecholamine-secreting tumors derived from the neural-crest tissue of the adrenal medulla or extra-adrenal paraganglia.^1^ The clinical presentation of these tumors is related to catecholamine production that can cause a constellation of nonspecific symptoms, often leading to misdiagnosis. The most commonly reported symptoms are headache, palpitations, diaphoresis, and hypertension.^1^ Nausea, weight loss, and pallor have also been reported.^1^ Hyperglycemia is also seen, as in the case of this patient, and cases of hyperglycemia as the initial presenting symptom of pheochromocytoma have been documented.^2^

The mechanism of pheochromocytoma-induced diabetes is related to insulin resistance and suppression caused by excess catecholamines.^2^ Through activation of hepatocyte β and α receptors, inhibition of insulin secretion in β cells of the pancreas, and blockade of GLUT4 transporters in muscle and adipose, catecholamines secreted in pheochromocytomas increase glycogenolysis and gluconeogenesis while interfering with normal feedback control of insulin.^3^

In a retrospective study of 191 patients with pheochromocytoma, diabetes was present in over 33% of the patients.^2^ These patients did not differ in body mass index compared to those in the study with pheochromocytoma without diabetes, and almost all patients were cured of their diabetes once their tumors were surgically removed.^2^ This case illustrates the importance of considering less common but potentially life-altering diagnoses such as pheochromocytoma in the patient with normal body mass index without a history of diabetes presenting with hypertension and hyperglycemia.

Secondary erythrocytosis due to overproduction of erythropoietin is rare but known to occur with pheochromocytomas and paragangliomas.^4-8^ In case reports, patients with paragangliomas and polycythemia have been shown to have gain-of-function mutations in the DNA coding for hypoxia-inducible factor proteins.^7,8^ The hypoxia-inducible factor proteins are transcriptions factors that upregulate EPO and associated genes and therefore directly cause the increase in erythropoiesis.^7,8^ This patient presented with a Hb of 17.8; however, this rapidly normalized with IVF administration and was therefore thought to reflect hemoconcentration in the setting of volume depletion rather than erythropoietin overproduction.

In summary, acute hyperglycemic crises are commonly encountered clinical scenarios faced by physicians. This case illustrates how pheochromocytomas, through catecholamine-induced stress responses, can present as hyperglycemic crisis. Keeping pheochromocytoma in the differential diagnosis will allow the astute clinician to notice the accompanying characteristics of a case that often unveil the presence of a pheochromocytoma.
